# Unraveling the Properties of Phage Display Fab Libraries and Their Use in the Selection of Gliadin-Specific Probes by Applying High-Throughput Nanopore Sequencing

**DOI:** 10.3390/v16050686

**Published:** 2024-04-26

**Authors:** Eduardo Garcia-Calvo, Aina García-García, Santiago Rodríguez, Rosario Martín, Teresa García

**Affiliations:** Department of Nutrition and Food Sciences, School of Veterinary Sciences, Universidad Complutense de Madrid, 28040 Madrid, Spain; edugar01@ucm.es (E.G.-C.); santro03@ucm.es (S.R.); rmartins@ucm.es (R.M.); tgarcia@ucm.es (T.G.)

**Keywords:** celiac disease, phage display, high-throughput DNA sequencing, nanopore sequencing, gliadin

## Abstract

Directed evolution is a pivotal strategy for new antibody discovery, which allowed the generation of high-affinity Fabs against gliadin from two antibody libraries in our previous studies. One of the libraries was exclusively derived from celiac patients’ mRNA (immune library) while the other was obtained through a protein engineering approach (semi-immune library). Recent advances in high-throughput DNA sequencing techniques are revolutionizing research across genomics, epigenomics, and transcriptomics. In the present work, an Oxford Nanopore in-lab sequencing device was used to comprehensively characterize the composition of the constructed libraries, both at the beginning and throughout the phage-mediated selection processes against gliadin. A customized analysis pipeline was used to select high-quality reads, annotate chain distribution, perform sequence analysis, and conduct statistical comparisons between the different selection rounds. Some immunological attributes of the most representative phage variants after the selection process were also determined. Sequencing results revealed the successful transfer of the celiac immune response features to the immune library and the antibodies derived from it, suggesting the crucial role of these features in guiding the selection of high-affinity recombinant Fabs against gliadin. In summary, high-throughput DNA sequencing has improved our understanding of the selection processes aimed at generating molecular binders against gliadin.

## 1. Introduction

The paradigm-shifting development of hybridoma technology revolutionized the field of antibody production with the discovery of monoclonal antibodies [1]. In 2022, six out of the fifteen best-selling pharmaceuticals were monoclonal antibodies (even more if COVID-19 vaccines are not considered); these figures illustrate the magnitude of the impact that these molecules have had in research and medicine [2]. Subsequently, the advent of recombinant protein production techniques and the application of directed evolution methodologies have further transformed the antibody discovery landscape [3].

Phage display technology stands as the most prominent methodology for the development of recombinant antibodies in the context of the directed evolution of proteins. This method is based on the construction of antibody libraries derived from diverse sources with high variability [4]. A characteristic feature of these libraries is that the antibodies are expressed fused to the coat proteins of bacteriophages, which facilitates their affinity selection through successive rounds against the target antigen (biopanning) [5].

Traditionally, monitoring of the panning procedure has been performed by colony picking and Sanger sequencing since the 1990s. However, while these methods can be automated for the screening of thousands of different colonies, they only cover a small amount of the clones implied in the process. This problem may be solved by using next-generation sequencing (NGS) techniques [6]. The in-depth analysis of phage libraries by NGS has proven to be an exceptionally effective approach for clone identification, including rare low-frequency variants that may not amplify adequately within the phage population [7]. Moreover, NGS has facilitated the identification and reconstruction of complementarity-determining region (CDR) combinations of antibody fragments from phage selection outputs [8]. Beyond antibody libraries, this transformative technique extends its applicability to the study of peptide libraries, essential for epitope mapping of antibodies [9].

Oxford Nanopore Technology offers many advantages that have revolutionized the field of DNA sequencing and genomic analysis. Firstly, the portable and compact sequencing devices provide unprecedented flexibility, enabling real-time, in situ, and rapid DNA sequencing in diverse environments, including field research and clinical settings. Secondly, the sequencing technology can read exceptionally long DNA fragments, allowing for improved genome assembly and structural variant detection. This feature has been particularly valuable for studying complex genomes or uncovering elusive genetic mutations. Additionally, Nanopore’s procedures eliminate the need for time-consuming library preparation, making them a faster and cost-effective option for researchers. Furthermore, they allow both DNA and RNA analysis, broadening their applicability in different research areas, from genomics to transcriptomics. Overall, the Oxford Nanopore sequencing technology represents a powerful tool for more comprehensive and efficient nucleic acid analysis [10].

Antibodies are lymphocyte-secreted molecules with the ability to attach to specific antigens and with several cell signaling functions. Their antigen-counteracting capacity derives from their extraordinary diversity; for instance, humans may be capable of producing as many as 10^18^ distinct antibodies [11]. This huge antibody repertoire that can be found in mammals derives from diverse genetic events like V(D)J recombination [12], somatic hypermutation, and class switch recombination [13].

In humans, the genes encoding antibodies are located on different loci found on three chromosomes (chr): heavy chain genes found on chr14 (14q32.33) and kappa and lambda light chain genes found on chr2 (2p11.2) and chr22 (22q11.2), respectively. To date, 50 germinal sequences have been found for VH (heavy chain variable region) grouped in 7 families, 40 for Vκ (kappa chain variable region) grouped in 6 families, and 30 for Vλ grouped in 10 families. The three major D gene fragments are located in the heavy chain locus. For the J genes, 7 genes have been described for the heavy chain locus, 9 for lambda, and 6 for the kappa locus. Moreover, IgHs (immunoglobulin heavy chains) have five major isotypes (IgM, IgD, IgG, IgE, and IgA), with four isotypes for IgG (IgG1, IgG2, IgG3, and IgG4) and two for IgA (IgA1 and IgA2) [14].

Several diseases related to the ingestion of gluten, the ethanol-soluble protein fraction present in the endosperm of some cereals like wheat, barley, and rye, have been described. The most studied is celiac disease, an autoimmune condition with a reported prevalence of 0.5–1% in the general population, although this might be underestimated due to undiagnosed or misdiagnosed milder cases [15]. This disease is characterized by the production of autoantibodies against tissue transglutaminase (tTG) in the context of a loosened intestinal barrier [16]. Despite ongoing research into numerous therapeutic alternatives for celiac patients, the gluten-free diet remains the most effective treatment, not only to halt intestinal damage but also to promote tissue healing [17].

In this work, two previously constructed antibody libraries, which were subjected to a biopanning process for the selection of gliadin-binding antibodies, were characterized by sequencing with Oxford Nanopore technology. A comparison of the changes in the compositions of the different antibody chains over consecutive rounds of panning provided an overall picture of the performance of the gliadin-guided selection procedure.

## 2. Materials and Methods

### 2.1. Source Material: DNA Isolation and Purification

In our previous works, high-affinity antibody fragments against gliadin were developed as novel probes for immunodetection systems to ensure the absence of gluten in foods intended for people suffering from gluten-related disorders. These antibodies consisted of antigen-binding regions (Fab) generated by phage display technology. For this purpose, two Fab libraries were constructed into the pComb3X vector [14]: an immune library derived from retrotranscribed RNA of the peripheral blood lymphocytes of celiac patients [18], and a second library obtained by merging semi-synthetic heavy chains (derived from a pre-existent single-domain antibody) with the immune light chain repertoire, termed the semi-immune library [19]. Both libraries were used in biopanning processes to select high-affinity Fab against gliadin. After each round of selection, the eluted phages were used to infect *Escherichia coli* XL1-Blue (*rec*A1 *end*A1 *gyr*A96 *thi*-1 *hsd*R17 *supE*44 *rel*A1 *lac* [F *pro*AB *lacIq*ZΔM15 *Tn*10 (*Tet*r)]) (Agilent©, Santa Clara, CA, USA, ref #200150). Cultures were grown in Super-Broth medium (SB: 30 g/L tryptone, 20 g/L yeast extract, 10 g/L MOPS, pH 7). After centrifugation at 4000× *g* for 20 min at room temperature, the supernatant was subjected to phage precipitation to continue the selection process, and phagemid DNA was isolated from the cell pellets through a midi-prep procedure (PureLink™ HiPure plasmid Midiprep Kit Invitrogen™, Thermo-Fisher©, Waltham, MA, USA, ref #K210004), following the manufacturer’s protocol for sequencing.

In this study, the unsearched libraries as well as the sub-libraries obtained after each round of panning were characterized by NGS with Oxford Nanopore technology. For this purpose, DNA in a double strain and linear format was required. Plasmids were digested with *Nhe*I for 1 h at 37 °C (New England Biolabs© Ipswich, MA, USA ref #R0156), since this enzyme presented a single restriction site with an appropriate distance from the antibody construct, to avoid interfering with the sequencing results.

The DNA was purified by AMPure XP reagent (Beckman Coulter©, Brea, CA, USA, ref #A63880) following the manufacturer’s instructions for a protocol based on the use of magnetic beads. Finally, DNA was quantified in a Qubit^®^ Fluorometer (Invitrogen, Thermo-Fisher©), and its quality was measured with a NanoDrop ND-1000 spectrophotometer (NanoDrop Technologies Inc., Montchanin, DE, USA).

### 2.2. DNA Preparation for Sequencing Protocol

A total of nine samples were barcoded with the PCR barcoding expansion 1–12 kit (Oxford Nanopore©, Oxford, UK, ref #EXP-PBC001) using a magnetic-bead-based protocol. Barcodes (BCs) were assigned as follows: BC01-06 for the identification of the un-panned immune library (BC01) and its four panning rounds (BC02–05) and BC06-09 for the identification of the un-panned semi-immune library (BC06) and its three panning rounds (BC07–09).

Finally, the magnetic bead system was used to subject the barcoded samples to end-repair and dA-tailing using the NEBNext End Repair/dA-tailing module, and we performed subsequent ligation of Nanopore sequencing adapters onto the prepared DNA ends (Oxford Nanopore©, ref #SQK-LSK110). Samples were loaded into the Nanopore flow cell (Oxford Nanopore©, ref #R10.4.1), and NGS sequencing was performed on a MinION^®^ device, collecting sequencing data for 30 h.

### 2.3. Primary Sequence Analysis

NGS sequence analysis usually follows a workflow with two main stages. The primary analysis is based on the organization of the raw sequences generated by the sequencer and the elimination of poor-quality sequences that could generate errors and introduce noise into the subsequent analysis.

MinKNOW 20.10 software (Oxford Nanopore©) transformed the registered electrical signals into raw FAST5 files that were used as the base for the primary analysis, which was carried out using a workflow encompassing three different steps. In the first step, the Megalodon tool (v1.9.5, https://github.com/nanoporetech/megalodon (accessed on 6 March 2023)) was used to extract high-accuracy modified base calls and sequence variant calls from the raw reads by anchoring the information of the basecalling neural network output to the reference genome of *Homo sapiens* (Genome Reference Consortium Human Build 38, NCBI RefSeq assembly GCF_000001405.26). A sub-pipeline appropriately divided the work and merged the results, yielding a single FASTQ file containing the basecalled reads. In the second step, this file was demultiplexed into new FASTQ files for as many barcodes as the analysis included, in order to cluster the sequences according to their barcodes. Subsequently, the files provided were submitted to quality control using FastQC and analyzed with the Porechop tool (https://github.com/rrwick/Porechop (accessed on 6 March 2023)) to trim off the adapters found at the ends of the reads. The trimmed sequences were again subjected to quality control, before being analyzed with Trimmomatic, a tool that can identify low-quality reads. Lastly, the trimmed sequences were subjected to a final quality control using FastQC. In the third step, a sorted BAM file was obtained with the alignment of the sequences against the reference genome of *Homo sapiens* using the minimap2 tool (https://github.com/lh3/minimap2 (accessed on 6 March 2023)).

The tools used in the secondary analysis required sequence packages containing fewer than one million sequences. For this purpose, the tool LUMC/fastaq filter (https://github.com/LUMC/fastq-filter (accessed on 7 March 2023)) was used to optimize the FASTQ packages, which were filtered by read size (between 4 and 6 kb, an appropriate size for the phagemids composing the library) and sequence quality (phred scores higher than 15, which means that the possibility of sequencing errors is less than 3%), removing low-quality sequences from the packages.

### 2.4. Secondary Analysis of Antibody Sequences

The filtered FASTQ data were uploaded to the online tool IMGT/HighV-Quest, which orchestrated the analysis of the Fab sequences [20]. Each identified Fab was characterized by (1) unique IMGT numbering for the V-domain; (2) closest variable (V), diversity (D), and junction (J) genes and their respective alleles; (3) junction analysis; and (4) mutation and amino acid changes resulting from somatic hypermutations, through comparison with the IMGT/V-QUEST reference directories set [21].

Subsequently, the IMGT/StatClonotype tool was used to perform a pairwise comparison of datasets from the IMGT/HighV-QUEST output. The following multiple testing procedures were applied: Bonferroni, Holm, Hochberg, Šidák single-tep, Šidák step-down, Benjamini and Hochberg (BH), and Benjamini and Yekutieli (BY). The rejection level used to discriminate whether the hypothesis was supported was 0.05 [22].

In this study, the different datasets corresponded with consecutive rounds of selection of the Fab phage libraries. We ascertained V(D)J gene associations according to multivariant phylogenetic heatmap plots, and we performed CDR analysis based on length and amino acid composition [23].

Minimap2 alignments were used to identify classes and subclasses of the antibody sequences that could not be analyzed by IMGT tools. The number of sequences that aligned at each position of the genome where the genes of each subclass were located was counted using the coverage function of the bedtools package (https://bedtools.readthedocs.io/en/latest/content/tools/coverage.html (accessed on 15 March 2023)).

## 3. Results and Discussion

### 3.1. DNA Preparation

Phagemid DNA isolated from all the library and sub-library samples resulted in high yields (DNA concentrations above 390 ng/μL) with appropriate purity (OD 260/280 > 1.8 and OD 230/260 > 2). As such, the obtained DNA samples showed suitable parameters for them to be subjected to the sequencing process.

### 3.2. Primary Sequence Analysis

After the transformation of the recorded electrical signal into nucleotide data (base-calling algorithm), a total of 4.68 million reads were collected in the FAST5 format. Data were collected in this format because it allows for efficient storing of both basecalled sequences and raw signal data. This is important when performing nanopore sequencing since raw signal data can provide additional information and enable more accurate basecalling or downstream analysis.

**Figure 1:**
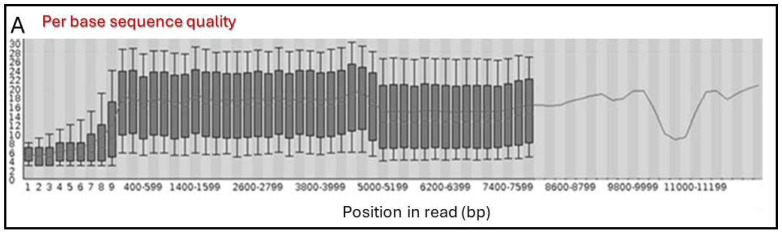

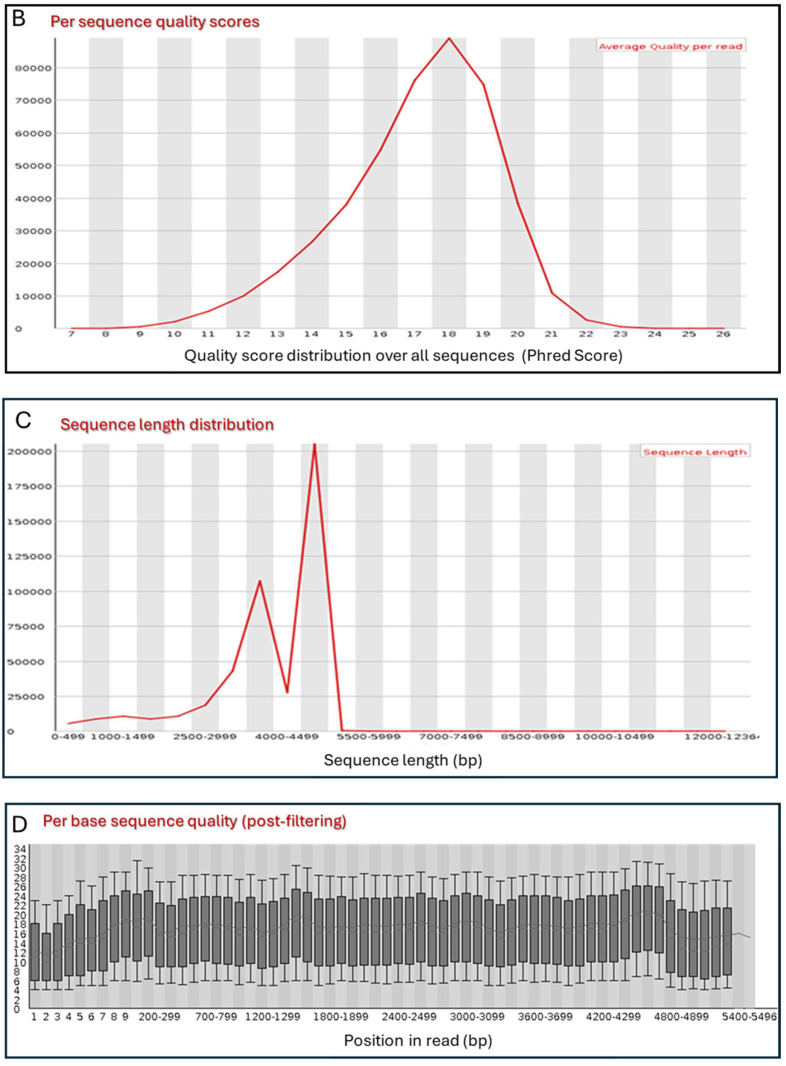
Quality metrics for Oxford Nanopore sequencing of the phage-displayed libraries. (**A**) Distribution of quality scores according to read position; in the boxplot, black lines represent medians, the boxes show the lower and upper boundaries of the first and third quartiles, respectively, and the lines show the mean quality scores. (**B**) Mean per-read quality score diagram. (**C**) Read length distribution. (**D**) Distribution of quality scores according to read position after sequence filtering, where black lines represent medians, the boxes show the lower and upper boundaries of the first and third quartiles, respectively, and the lines show the mean quality scores. All the analyses were performed using the FastQC toolkit. The data shown correspond to one of the multiplexed samples; however, the data recorded for all the samples presented similar profiles.

Through the primary analysis workflow, the FAST5 reads were transformed into demultiplexed and trimmed FASTQ sequences grouped by barcode. An average of 0.5 million sequences per barcode were collected, ranging in length from 120 to 12,400 bases, which is common in this methodology [24]. This broad spectrum of reads underscored the imperative need to implement a filtering process. The quality of sequences was assessed and showed a relatively uniform distribution across the reads (Figure 1A). The analysis of quality scores’ distribution per sequence revealed that the majority of sequences exhibited a phred score of 18 (Figure 1B), demonstrating an improvement in quality over those reported by previous similar studies, where most of the sequences presented a phred score of between 10 and 15 [25]. The examination of the sequence length distribution revealed the presence of two prominent populations. The larger population, obtained in lengths ranging from 4.5 to 5.5 kb, corresponded to the linearized phagemid of the library (Figure 1C). On the contrary, the smaller population centered on 4 kb, could potentially denote truncated plasmids as a result of unforeseen restriction points derived from the variability of the antibody sequences or the deletion of plasmid segments known to arise in *E. coli* [26]. Based on the quality data described above, sequence filtering was performed, obtaining a collection of cleansed FASTQ sequences of 4–6 kb in length and phred scores greater than 15. The increment of the quality of the sequences after filtering can be observed in Figure 1D. Remarkably, the majority of the antibody coding sequences were found to meet these parameters and were, therefore, included in the secondary analysis; other sequences with varying lengths were considered noise or outliers and were excluded from the analysis.

### 3.3. Secondary Analysis of Antibody Sequences and Biological Interpretation of the Results

After completing the primary analysis and acquiring a set of adequate sequences in terms of quality and length, subsequent analysis delved into the antibody composition of the constructed libraries.

Firstly, all the sequences were aligned to the V(D)J germlines using IMGT/HighV- Quest. The immune library data were analyzed for both the heavy and light chains, whereas, for the semi-immune library sequences, only the light chains were analyzed since in this case all heavy chains derived from the same dAb (VH3-23 subfamily), and their study was not relevant.

In this study, 31 out of the 50 VH subfamilies documented in the human genome were captured in the immune library. Regarding the light chains, 16 Vκ subfamilies were successfully captured within the immune repertoire, and 22 Vκ subfamilies within the semi-immune repertoire, from the total of 40 Vκ subfamilies known in humans [14]. It should be noted that not all antibody chains were included in the analysis, primarily due to the PCR amplification conditions when the library was built. Our primary objective was to focus on including antibody chains specifically involved in the immune response associated with celiac disease as reported by other authors [27,28].

Moreover, it is worth noting that our decision not to include all antibody chains aligns with findings from previous studies on repertoire construction for phage display, which indicate that including many minor subfamilies in repertoires does not necessarily add practical value, as such subfamilies are often compromised and do not significantly contribute to the functionality of the repertoires [29]. Therefore, in the present study, a more strategic approach focusing on the amplification of genes known to code chains that exhibit reactivity against gliadin (subisotypes IgA, IgG1, and IgG2) was undertaken. This strategy was supported by the humoral response obtained from the serum samples of celiac donors used for the amplification of antibody-coding genes [18].

#### 3.3.1. Analysis of the Variable Domains

Regarding the VH composition of the immune library, two different profiles could be observed during the panning rounds. First, the profile of the 31 VH subfamilies between the unpanned library and the phage population recovered from the first round of selection remained practically unaltered. Then, a slight increment of the VH3-15 subfamily was observed after the second round, followed by a substantial dominance of this subfamily in rounds three and four. These data are in agreement with those obtained in our previous work by Sanger sequencing, in which the complete dominance of the VH3-15 subfamily was also observed in the last round of selection [18]. Moreover, the high affinity of these VH3-15 Fabs for the target antigen (gliadin) was demonstrated both in their phage format [18] and with soluble Fabs [30]. This supports the hypothesis that the selection process was affinity-driven and that the obtained sequences do not derive from non-specific phage expansion or sequencing artifacts.

**Figure 2:**
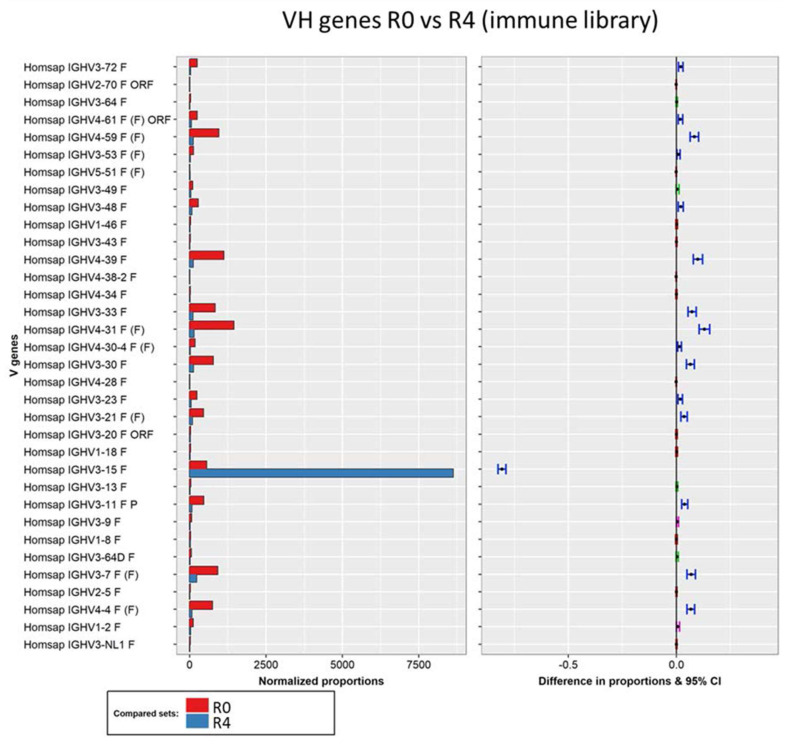
Pairwise analysis of VH gene composition of the unpanned immune library (red) and its fourth round of selection (blue). Normalized proportions (left chart) and 95% confidence intervals of the differences in proportions (right chart) for each VH subfamily. The color of the intervals depicts test interpretation: non-significant (red), significant for all p-values (blue), significant for at least 2 tests (pink), and significant for only the Benjamini–Hochberg test (green).

In order to test this hypothesis, pairwise comparisons of the heavy chain composition between the different rounds were conducted using IMGT/StatClonotype. To study the selection process, the unpanned immune library and its fourth round of selection were compared to observe any changes in the distribution of the V gene. As expected, there was a significant increase (statistically significant for all *p*-values) in the VH3-15 subfamily, which evolved from a minority to a predominant family (Figure 2). Although less pronounced, there was also a statistically significant decrease in the presence of 15 subfamilies, with the remaining subfamilies showing no notable differences.

**Figure 3:**
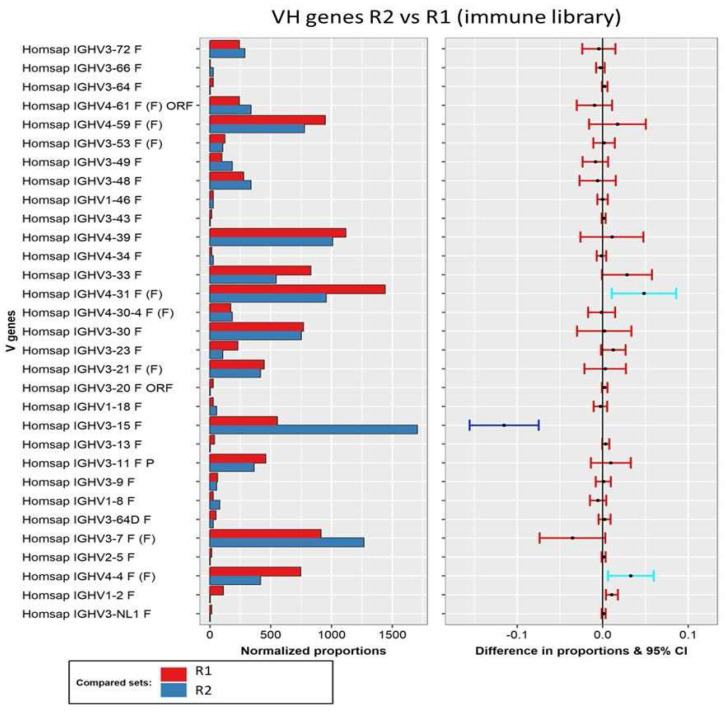
Pairwise analysis of VH gene composition of round 1 (red) and round 2 (blue) of selection of the immune library. Normalized proportions (left chart) and 95% confidence intervals of the differences in proportions (right chart) for each VH subfamily. The color of the intervals depicts test interpretation: non-significant (red), significant for all p-values (dark blue), and significant for one test (light blue).

Additionally, we also explored through pairwise analysis whether the initial increase in the VH3-15 subfamily between rounds 1 and 2 was significant (Figure 3). This test confirmed that the VH3-15 subfamily exhibited the most substantial increase, which was statistically significant for all *p*-values analyzed. Additionally, two VH4 subfamilies (VH4-4 and VH4-31) showed a significant decrease, although this significance was observed for only one of the p-values and these subfamilies were not highly represented in the last round of selection.

When examining the selection process of the immune library, a striking observation was made. Instead of the anticipated gradual increase in selected binders across the rounds, two punctual selection events were identified. The first event was characterized by a slight yet significant increase in the VH3-15 Fab variants during the second round. This was followed by their substantial expansion in rounds 3 and 4. Notably, this event coincided with an increase in the stringency of the selection, achieved by incorporating additional washing steps. Following the expansion observed in round 3, the VH3-15 variants surpassed the abundance of other variants to such an extent that the likelihood of other selections became highly improbable. Although there were several notable changes in the composition of the light chain families throughout the depicted process, it appears that the role of the light chain as a selection agent may not be decisive.

**Figure 4:**
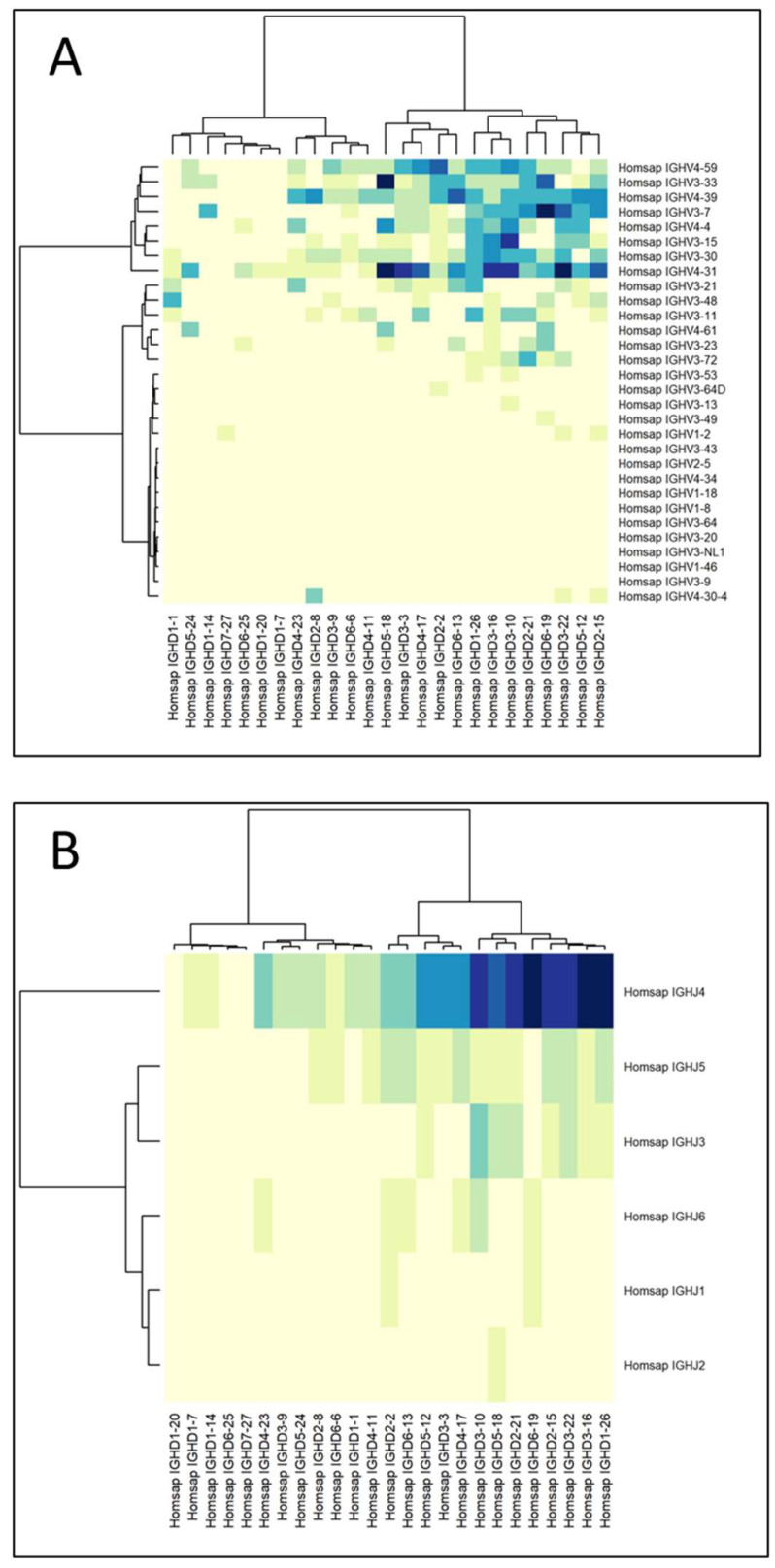

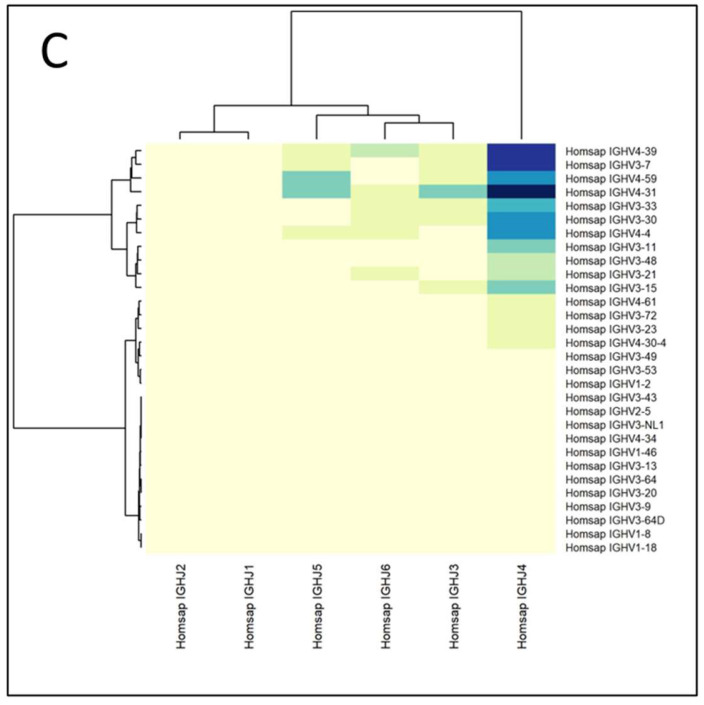
Clustered image maps depicting the relations between immunoglobulins’ VDJ genes of the immune library. (**A**) VD genes, (**B**) DJ genes, and (**C**) VJ genes. Darker colors on the heatmaps indicate the strongest associations between genes.

The relationship between the VDJ genes of the heavy chains of the immune library was depicted by generating clustered image maps for the identified VD, DJ, and VJ genes (Figure 4). Considering that the rearrangement of the chains did not change through the selection rounds, the analysis was performed using data from the unpanned library. The main gene associations found were as follows: VH3-33 with DH5-18 and JH4; VH4-31 with DH5-18, DH3-22, and JH4; VH3-7 with DH6-19 and JH4; VH3-15 with DH3-10; and JH4 with DH6-19, DH3-16, and DH1-26. Notably, the usage of the joining gene JH4 was very extended, a normal pattern in human species where JH genes present an asymmetric usage of more than 40% for JH4. This situation is derived from rearrangement events, in which conserved nonamers, heptamers, and 23 bp spacers are involved [31]. VH3-15 pairs with DH3-10 and JH4, resulting in elongated HCDR3s, as observed in the gliadin-reactive Fabs obtained from the final round of selection [18]. In addition, the molecular docking of these Fabs suggested that their large HCDR3 could potentially interact with various regions of the antigens through both sides of the loop, leading to stronger interactions. This observation could explain the extremely specific selection of these VH3-15 Fabs during the biopanning rounds.

Another crucial aspect under investigation pertains to the identification of specific characteristics associated with the celiac response within the immune library. Notably, it was observed that the selected chain variants (VH3-15) constituted approximately 3.5% of the total reads during the initial round. It should be highlighted that the presence of the VH3-15 subfamily was notably higher than that reported in other human naïve phage display libraries [32]. In the present study, these variants were selected until the total dominance was reached of the population in the last round of biopanning (86.2%).

Previous studies have also emphasized the preferential selection of VH3-15-IgA variants as highly reactive antibodies against gliadin [33]. However, it is worth noting that the naturally occurring VH/VL pairing was not consistently maintained in our study, as this phenomenon occurred stochastically during the construction of the library.

An additional analysis was performed focused on the selected Fab variants from the immune library that demonstrated a high affinity for gliadin and good performance in tests for gluten detection in foodstuffs in previous works [18,27]. These variants were clustered in 17 clonotypes belonging to subfamily VH3-15. The sequences of these variants were aligned against the germline gene (IMGT accession number M99406), revealing several mismatches that could constitute biologically significant mutations in the gliadin-binding Fab (Table 1).

Upon analyzing the selected variants, a comprehensive examination revealed the presence of somatic mutations within the VH regions. Specifically, an average of 9 ± 2 mismatched bases were identified across the VH3-15 variants from the final selection round. Remarkably, these mismatches were distributed extensively throughout the reads, indicating a strong likelihood of somatic hypermutation (SHM) as the underlying cause. By analyzing the effects of the observed DNA mismatches on amino acid translation, it was found that two of them (positions 150 and 252) were silent mutations that did not result in changes in the amino acid sequence. In contrast, the remaining seven mismatches were classified as point mutations. Among them, the mutations 99 and 176 could lead to significant alterations in the amino acid composition, with one causing a substitution from an aromatic group (W) to a polar non-charged residue (C), and the opposite occurring in the other (T into F). The point mutations 13, 15, 92, 188, and 193 involved changes between similar amino acids (e.g., hydrophobic to hydrophobic residues at position 13). From a structural perspective, only three of these point mutations were found within the CDRs, specifically, the positions 92 and 99, which correspond to CDR1, and the position 176, corresponding to CDR2.

In our study, we observed a lower occurrence of mutations in the selected IgA Fabs. This observation has also been reported in other studies, where it was depicted that serum antibodies reactive to gliadin presented only an average of 9 mutation on their VH genes, while the control IgA antibodies exhibited an average of 15 mutations [33].

This low occurrence of SHM observed in the celiac-patient-derived immune library is typically found in antibodies derived from T-cell-dependent extrafollicular activation of B cells [34] and short-lived germinal center responses [35]. Moreover, extrafollicular responses typically result in the generation of short-lived plasma cells and a rapid decrease in serum antibodies following the immune response [36]. These events underline the importance of selecting patients who had not initiated a gluten-free diet for the construction of the library. Without exposure to gluten-related response-triggering peptides, these short-lived plasma cells would have been suppressed, potentially leading to the exclusion of genes encoding gliadin-reactive antibodies in the library.

In addition, the development of B cells in the extrafollicular context is influenced by T cells. Previous reports have indicated that T cells present at the T-B border within lymph nodes, distinct in phenotype from germinal center T cells, play a crucial role in the initiation of B-cell priming for the generation of extrafollicular antibody responses [37]. This lower mutation occurrence in gliadin reactive antibodies was also found in transglutaminase 2 (TG2) autoantibodies, suggesting that the presence of gluten antigens or gluten-specific T cells may potentially contribute to the observed low mutation rates in the specific IgA antibodies [33].

**Figure 5:**
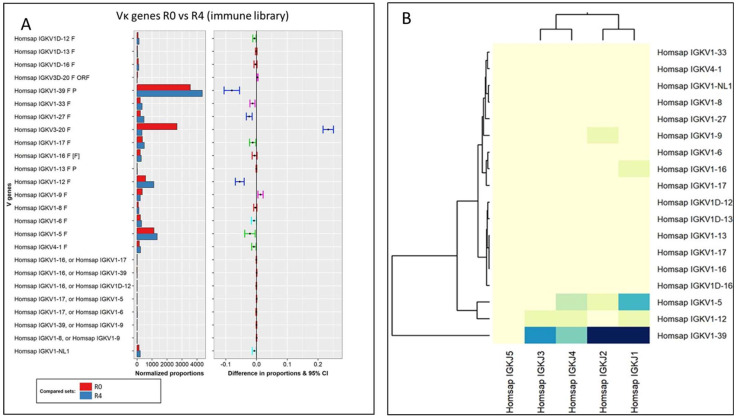
(**A**) Pairwise analysis of Vκ gene composition of the unpanned immune library (red) and its fourth round of selection (blue). Normalized proportions (left chart) and 95% confidence intervals of the differences in proportions (right chart) for each VH subfamily. The color of the intervals depicts test interpretation: non-significant (red), significant for all *p*-values (dark blue), significant for one test (light blue), and significant only Benjamini–Hochberg test (green). (**B**) Clustered image map depicting the relations between VJ (κ chains) genes of the immune library. Darker colors on the heatmap indicate the strongest associations between genes.

When analyzing the evolution of the immune library kappa-light chains throughout the selection process (unpanned library against round 4), four statistically significant changes were found (Figure 5A): increases in Vκ1-39, Vκ1-27, and Vκ1-12 and a decrease in Vκ3-20 subfamilies. In this sense, Vκ1-39 has been identified as the most expressed light chain gene in other studies with naïve libraries [32]. In the present study, this light chain subfamily was not only found to be dominant in the unpanned immune library but also between the selected phages after the panning process (the subfamily showing the most significant increase within the light chains).

Regarding the rearrangement process for the kappa-light chains, several VJ associations were found between the most expressed Vκ gene (Vκ1-39) with Jκ-1 to 4, with a stronger occurrence of Jκ-1 and Jκ-2 associations (Figure 5B). Although there were several notable changes in the composition of light chain families throughout the immune repertoire selection process, it seemed that the role of the light chain as a binding determinant was not decisive for the enrichment.

In summary, the immune libraries and their selection process successfully captured and preserved characteristics associated with antibodies found in the celiac disease response, as identified by multiple researchers [27,28]. These features were utilized to generate a novel high-affinity Fab targeting gluten. The resulting Fab possesses unique attributes, incorporating key elements of the humoral response observed in celiac patients like a lower appearance of SHM, which demonstrates that the libraries incorporated antibody chains expressed by gliadin-reactive B cells activated by an extrafollicular stimulation. Consequently, they serve as valuable probes for gluten immune detection in food products. This approach offers distinct advantages over the traditional monoclonal-antibody-based methods that rely on animal immunization, which are currently available in the market.

**Figure 6:**
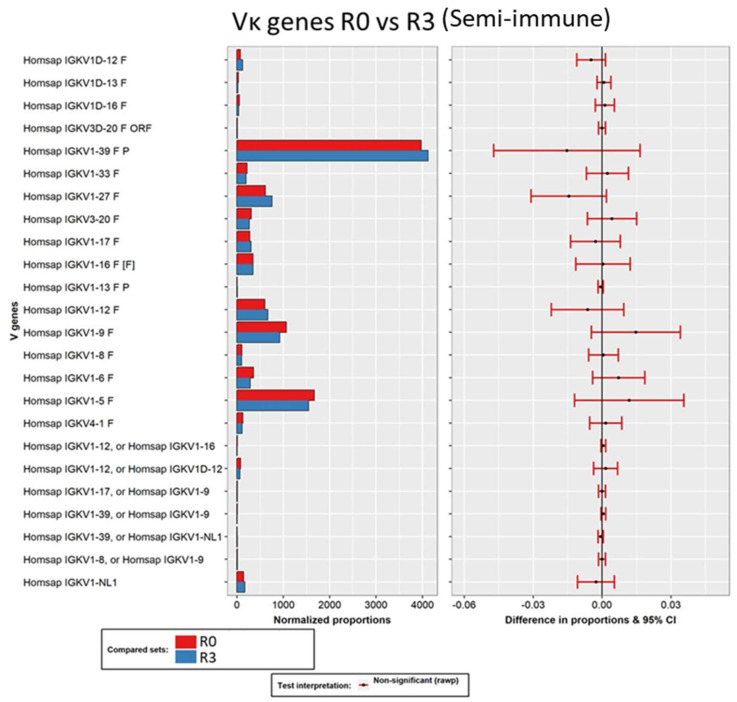
Pairwise analysis of Vκ gene composition of the unpanned semi-immune library (red) and its third round of selection (blue). Normalized proportions (left chart) and 95% confidence intervals of the differences in proportions (right chart) for each VH subfamily. The color of the intervals depicts test interpretation: non-significant (red).

Similar analyses were performed with the data obtained from the sequencing of the library constructed by merging immune and semi-synthetic chains, but mainly focusing on the light chains. In this case, no significant changes in the composition of the light chains were found through the selection process (Figure 6). Since this library was built based on a pre-existent dAb (previously isolated for its affinity for gliadin), which was present in all the sequences, the result demonstrated that the immune light chains were not a determinant selector of gliadin-binding Fab.

#### 3.3.2. Analysis of the Constant Domains

**Figure 7:**
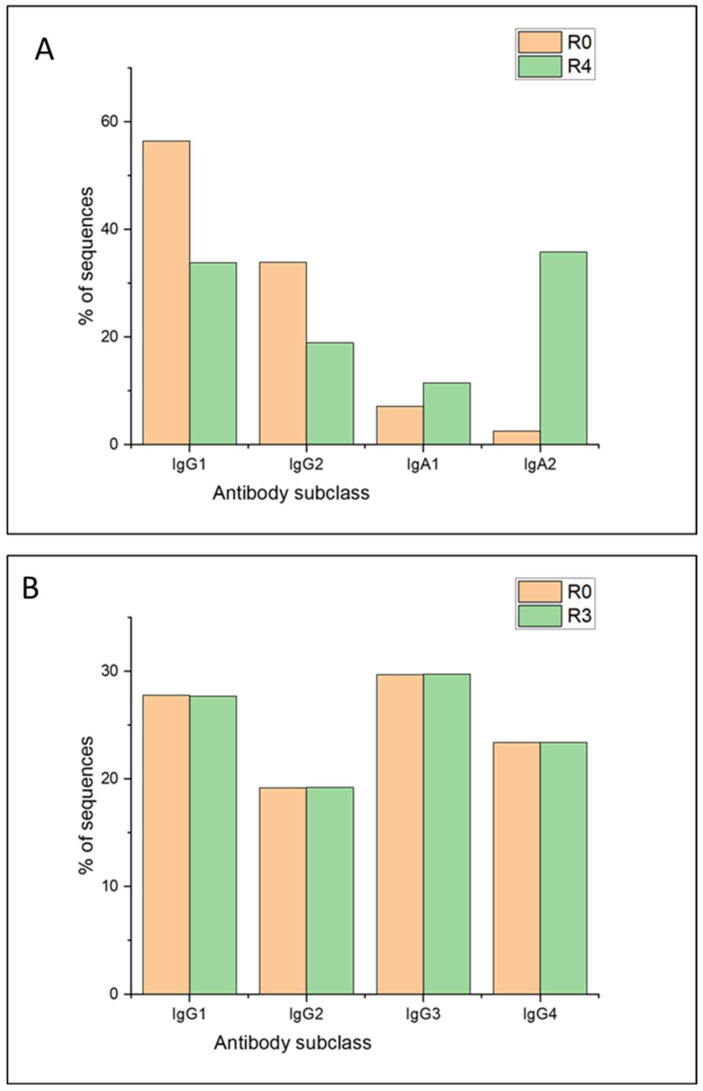
Antibody subclass composition of the unpanned immune (**A**) and semi-immune (**B**) libraries (R0, orange) and their last round of selection (green). Data were extracted from the alignment of the sequences of each round against the human genome, followed by counting sequences with the coverage function of the bedtools package.

An additional analysis, not commonly implemented in this kind of study, involved the identification of changes in antibody classes and subclasses that might occur during the selection process. This analysis is of particular importance in the characterization of the immune library since it may contribute to demonstrating the translocation of the humoral response found in celiac donors, in the phagemid DNA that constitute the library. This study revealed a clear change in the antibody subclass when comparing the unpanned library and its fourth round of selection (Figure 7A). Before the selection process, the vast majority of chains presented an IgG1 subclass (56%), followed by 33% with an IgG2 subclass, and 10% with IgA chains. On the other hand, after the selection process, the most represented chain was IgA2 (36%), which resulted in the minority subclass in the unpanned library (14-fold increase in abundance). As observed, the composition of antibody classes and subclasses completely changed during the selection process against gliadin. This finding further correlates with the fact that IgA2 was the subclass of the best gliadin-binding Fab isolated from the library [18].

The library composition by subclass, before the selection process, closely resembled that of IgG in human serum, where IgG represents over 80% of immunoglobulins. Furthermore, the customary 2:1 ratio in the quantity of IgG1 and IgG2 was maintained [38]. The proportion of IgA antibodies in the repertoire also mirrored the typical range found in healthy human serum, which is approximately 10–15% [39]. However, it is important to note that in celiac disease, this proportion is often increased, as reported in previous works [15], a phenomenon that was not captured in our repertoire.

Notably, there was a clear selection of antibodies from the IgA2 subclass. IgA2 is minimally represented in the bloodstream under normal circumstances, except in cases of intense celiac-type responses, where antibodies from this subclass (typically expressed in the intestinal mucosa) appear in the plasma [40]. Therefore, it is not surprising that the most reactive antibodies against gliadin that were selected predominantly belonged to the IgA2 subclass. In addition, no significant changes were observed in the comparison of antibody subclasses between the unpanned semi-immune library and its last round of selection (Figure 7B).

Although equimolar PCR products were used in the library construction procedure with the intention of achieving equal representation of the heavy chain subclasses (25% each), sequencing data indicated that certain chains had been amplified more efficiently than others, resulting in a slight imbalance.

In the present study, the investigation of the merged library revealed an absence of discernible evolutionary dynamics. Notably, no substantial alterations were observed between the initial and final rounds of selection for the light chains. This trend was similarly observed for the heavy chains, where the IgG3 Fab exhibited a high level of recurrence with nearly identical frequencies at both endpoints. The preservation of dAb functionality while transitioning into Fab can be attributed primarily to structural factors, specifically the transformation of the heavy chain and the incorporation of immune light chains, as previously demonstrated by ELISA experiments and computational models [19]. Consequently, it can be postulated that the acquisition of diverse antibody variants through evolution and selection processes played a lesser role in this particular context.

## 4. Conclusions

This study presents an illustrative demonstration of a rapid and precise methodology for sequencing and analyzing antibody repertoires (and their usage) generated through phage-mediated affinity-selection processes. The utilization of Oxford Nanopore technology facilitated a user-friendly and expeditious sequencing approach using portable equipment. This compact device enables the acquisition of significantly longer reads, thereby allowing simultaneous sequencing of the entire Fab, obviating the need for assembly from fragmented reads. The proposed workflow analysis enabled the generation of valuable data for a comprehensive characterization of the selection process, revealing features that may be overlooked by other analytical methods, such as traditional Sanger sequencing of individual colonies. This approach provides a deeper insight into the selection process, capturing additional information that would otherwise be inaccessible due to the size of the data generated.

The primary analysis approach facilitated a rapid and seamless handling of the raw data produced by the sequencing device. This encompassed key steps such as basecalling utilizing neural networks, demultiplexing, quality analysis, trimming low-quality sequences, and alignment against the reference genome. These steps effectively prepared the data for secondary analysis focused on antibody data generation, utilizing only high-quality sequences.

The secondary analysis involved the utilization of bioinformatics tools, specifically IMGT/HighV-Quest, for the classification of reads based on VDJ genes, CDR features, and clonotype organization. Additionally, IMGT/StatClonotype was employed for statistical analysis, and alignment-dependent analysis was conducted for isotyping and mutation analysis. These bioinformatics tools provided essential functionalities to enable comprehensive exploration and interpretation of the obtained data in an immunological context.

The progression of the panning process of the immune library against gliadin revealed a discernible selection pattern, as evidenced by the substantial expansion of VH3-15 variants within the Fab population starting from round 2, despite the initial presence of over 30 heavy chain families. While there were notable alterations observed in the composition of light chains, the data suggest that these chains did not play a significant role in the depicted selection process.

The analysis specifically targeting the merged chain library revealed that the conservation and expansion of features observed in the transformation of a pre-existing dAb into a Fab were not dependent on molecular selection. Significantly, no substantial changes were detected in the composition of antibody chains, suggesting that other factors such as structural characteristics of the Fab might have contributed to the gliadin-binding Fabs isolated when compared to the parental dAb.

Finally, the analysis of the VH3-15 variants that were selected during the panning process provided insights into certain immunological characteristics of the Fab. The lower observed mutation rate in the VH segment, consistent with previous findings [29], suggests that the generated Fab originated from plasma cells activated through T-cell-dependent extrafollicular stimulation. These identified features validate our proposal that the immune library generated encompasses crucial attributes transferred from the humoral response of individuals with celiac disease.

## Figures and Tables

**Table 1:** Identification of point mutations heavily repeated on the selected Fab variants from the immune library after the biopanning process against gliadin.

Position	Germline	Library	% of Reads with the Mismatch	Amino Acid Changes
13	G	C	92	V → L
15	G	C	91	V → A
92	A	G	91	N → T
99	G	T	93	W → C
150	T	G	96	R → R
176	C	A	91	T → F
188	C	G	93	A → G
193	C	T	95	P → S
252	A	G	96	Q → Q

## Data Availability

Data is available upon request to the authors.
